# Lectures on the Principles of Surgery; with Notes

**Published:** 1838-01

**Authors:** 


### ART. III.

*The Works of* JOHN HUNTER, F.R.S.; *with Notes.* Edited by J. F. PALMER, &c. Vol. I.—*London*, 1835. 8vo. pp. 643.

*Lectures on the Principles of Surgery; with Notes.* By JAMES F. PALMER, Senior Surgeon to the St. George's and St. James's Dispensary, &c.

AFTER the lapse of almost half a century from the death of John Hunter, we are for the first time presented, by means of the present publication, with his Lectures on the Principles of Surgery. They constitute a second division of the first volume of the new edition of Hunter's Works; and, from the facts stated by the editor, no doubt will be entertained of their being perfectly genuine. They are printed, we are informed in the preface, from a very full and accurate copy taken in short-hand by Mr. Henry Rumsey, of Chesham, which the editor has compared with certain other copies extant, and by the aid of which he has in some instances been enabled "to retrench redundancies and repetitions, and to substitute one word for another having a synonymous expression." However, he has never, by this proceeding, interfered with the true Hunterian character of the style, distinguished as it always is by plainness and vigour of expression, and by a strain of singular candour and honesty in the record of experiments or in the statement of pure matters of fact.

We have perused these Lectures with no ordinary feelings of satisfaction. They embody an immense amount of important facts, directed with no common skill to the illustration and improvement of medical science generally, and of the surgical department in particular. Indeed, we have no hesitation in saying that, whatever be the position of the reader in the profession, he will not relinquish the perusal of these Lectures, without the consciousness of having usefully employed the time which he may have bestowed upon them. For they constitute, in the fullest sense of the term, a philosophical disquisition on the science of Surgery; and hence, embracing the great principles on which the whole art of healing rests, their interest will be felt by all who regard Medicine as a true branch of science, and who delight to witness the gradual development of principles in the right interpretation of the phenomena of nature.

What tends to give to the present work so much of a *general* interest is the systematic manner in which the author proceeds in the discussion of his subject: commencing by brief explanations of the simplest facts of nature, he leads the student progressively onwards in the consideration of living phenomena, and the mind of the reader is thus gradually brought to the contemplation of the bearings of general laws upon the particular subject to which his attention is especially solicited. And, in this respect, in expounding the principles of surgery as a branch of natural philosophy, the Lectures of Hunter possess distinguishing superiority. They may not be well adapted for a Manual of reference, or as a mere Vade Mecum to the practitioner; but, as supplying him with a solid groundwork in the scientific principles of the profession, they constitute, we conceive, a very important addition to the medical literature of our country. It is quite true that objections will, in the present day, apply to some of the doctrines advanced in them; but the same result happens to everything that is written upon a science of experience, which, being essentially progressive, is constantly receiving additions and improvements. And yet there are works, in every department of natural science, that always maintain a certain standard value, from their intrinsic excellence, and are never superseded entirely by newer contributions. In this class we are sure that Hunter's Surgical Lectures must ever command a place. The principles of surgery, as known in his day, are so clearly and fully explained; the immense improvements, wrought by his own almost unaided energies, are so forcibly impressed upon the reader's attention; and the rigorous exactness of the observations and experiments recorded is so admirably established in their relation to principles,—that we can entertain no doubt that these Lectures will long be regarded as an important and valuable storehouse of information, not only by those who are engaged in the actual study or practice of our profession, but by all who are interested in the progress of science in general.

The subject of these Lectures, which occupy twenty-three chapters, naturally suggests a division of their contents into three portions,—physiological, pathological, and practical. The lectures purely physiological are comprised in eight chapters; and when it is remembered that John Hunter was the first who systematically applied the principles of physiology to the elucidation of surgical diseases, his views in this department must possess much interest. We are sorry that our limits will only allow us to notice a few of the more interesting points here discussed, and this only in the most cursory manner.

Hunter devotes a whole chapter to the exposition of his ideas upon the "Vital Principle;" and, as it is well known that a large proportion of his time was given to the elucidation of this subject, his detailed views, though not always in accordance with those which are now most generally recognized, will be found to possess a high degree of interest. The vital principle, with Hunter, is reducible, in all animals, to one simple element, being in itself either something actually superadded to the matter which it vivifies, or an essence arising out of the material arrangement under which life is manifested. The evidences of the endowment of any combination of matter with this principle are the power of self-preservation, and the power of action: these, though arising from the same source, are very different properties, as the first may exist independently of the second: thus, a fresh egg, though alive, has no vital action, and yet, even prior to incubation, it maintains a very considerable power of self-preservation, and of resistance to ordinary decomposing agency. Life, then, Hunter contends, is not *action;* though it is continued and supported by action, when this is once set up. Thus, in the higher classes of animals, "if the heart acts, the lungs must fulfil their part; the stomach must digest, the other parts subservient to this organ must be put in motion, and the secretory organs, nerves, and voluntary muscles." In this manner, although such a series of movements was not requisite to constitute the simple presence of life, yet, having once commenced, their absolute suspension in any combination of animal materials destroys the vital principle. He maintained that this principle was one simple property in every animal; and he certainly felt the disposition, common to his own, and more especially to the preceding age, to generalize too hastily the phenomena indicative of life, and almost to personify their occult cause as an independent existence. However, when, in any investigation, he proposed to himself to rectify notions which he believed to be erroneous, or to elicit some new truth, he never proceeded upon any such speculative grounds, but boldly interrogated nature by direct experiment. Indeed, we consider that the period in which Hunter flourished may not inaptly be regarded as the transition era of medical science,—as that in which the speculative was exchanged for the inductive philosophy, as the basis of research; and we have alluded more particularly to the hypothetical notions concerning the vital principle, as an illustration of the influence which mere speculation yet retained, even over minds of the most powerful order. For no one acquainted with the history of Medicine will fail to recognize, in the abstract vital principle of Hunter, the lingering shades of the *Archæus* of Van Helmont, or of the *Anima* of Stahl. Of life, beyond its observable effects, we can obviously know nothing; and hence, in the present day, we investigate only the conditions under which it is manifested, and enquire not what are its absolute laws, nor what is its actual essence.

The third chapter is devoted to the consideration of the Physiology of the Blood, which, as all are aware, had generally, before Hunter, been regarded, in his own words, "as a passive, inanimate, moving fluid, found everywhere in the body, deriving motion from the heart for the various purposes of the whole, then returning to the heart to be sent out again." In treating of this subject, Mr. Hunter explains and illustrates his own views regarding the vitality of this fluid. This is done in a very concise and analytical manner; and upon the original notions, propounded on this matter, will always depend a large share of Hunter's well-earned fame. It must also be admitted that even although the idea of the life of the blood should be questioned, the views maintained by him have led to results of practical importance, both in surgery and medicine.

In Chap. iv. we have a short sketch of the Principles of Organization and Action. The first is regarded in a point of view somewhat too mechanical to harmonise well with the doctrines of the present day, as the following extract will evince.

"Solidity, in a certain degree, is necessary for self-motion; for parts cannot pro-

LIBRARY OF THE

Orleans Parish Medical Society duce motion in one or other without some resistance or fixed point of motion. We therefore find the acting parts of an animal composed of solids, or the parts which compose them could not coalesce together by the attraction of cohesion; and it is necessary that it should be so, as without this no determined action could be produced.

"Now we have gone so far with the materials of an animal, let us next examine how these materials are disposed so as to form an animal. These materials may now be considered in a mechanical point of view, like the component parts of a machine, each of which has its destined use and own peculiarity of form. These are united with each other to form parts, the whole forming organs of various kinds to produce the mechanical effects required. These organs, again, united according to certain established rules, form animals. This compounding of animal matter is what should be understood by organization. Now, if this idea of organization is just, organization and life are two different things; for, according to this definition, a dead body is as much organized as a living one, for in the dead body the same mechanism exists as in the living one.'' (P. 241.)

We cannot assent to the proposition that "this compounding of animal matter is what should be understood by organization." By the term organization we understand an association of particles of matter in certain mechanical, chemical, and *unknown* relations, and any statement that would define organization merely upon the principles of mechanical or chemical philosophy must always be inexact, if some condition, not yet understood, be not implied in the definition. But herein we have another instance of Hunter's adhesion, in some respects, to the imperfect views of a preceding generation, and certain remnants of the doctrines of the chemical and intro-mathematical schools of the seventeenth century will be traced in this explanation of the nature of organization. When he proceeds, however, to develop his ideas regarding the "actions of animals," he manifests, in a high degree, the triumph of his vigorous and matter-of-fact understanding over the mere opinions and partial views of his own and preceding times, and briefly describes many of the functions of the animal economy, in a manner almost entirely compatible with all that has been revealed by modern investigation and research.

The "Brain and Sensation" constitute the subject of a separate chapter; and herein we have a summary of the state of information in the department of physiology, as it existed in Hunter's day, without any very striking illustrations. The whole of this matter is, however, so extensive, and the advance that has been made within a recent period is so considerable, that it would be impossible, within our limits, to furnish any epitome of Hunter's views, or to attempt any parallel between the existing condition of our knowledge upon the subject and its state in his own day. We must content ourselves with referring the reader to the volume itself.

We can do little more than mention the title of the next division of these lectures: "Of Susceptibility of Impressions; of Stimuli; of Dispositions of the Body; of Habit and Custom." The notions advanced under these heads are almost altogether theoretical; and, as will readily be conceived, are not such as are best calculated to indicate the powerful and energetic character of the mind from which they emanated. It was in dealing with the facts of nature, capable of actual demonstration by repeated and varied experiments, that Hunter's genius displayed itself; and in the ensuing chapter we have presented to us a subject more congenial to his mental habits, that of Animal Heat, and one upon which he threw much light by his investigations.

Hunter rejected all merely chemical or mechanical modes of accounting for the production of animal heat, and anticipated the law, recently established in the most complete manner by the experiments of Dr. Edwards, that the animal power of generating heat increases as its external presence is withdrawn. The following experiment, though undertaken with another object, first suggested this to his mind: besides its inherent interest, it is somewhat amusing from the simplicity with which he announces the failure of a grand speculation which he had entertained, and to which we referred in the account of his Life in our last Number.

"In the year 1766, two carp were put into a glass vessel with common river water, and the vessel was put into a freezing mixture. The water surrounding the fish froze very rapidly on the inside of the glass all round. When the freezing process approached the fish, it became as it were stationary; and the remaining water not freezing fast enough, in order to make it freeze sooner I put in as much cold snow as made the whole thick. The snow round the carp melted. I put in more snow, which melted also. This was repeated several times, till I grew tired, and I left them covered up to freeze by the joint operation of the mixture and the atmosphere. After having exhausted the whole power of life in the production of heat, they froze; but that life was gone could not be known till we thawed the animals, which was done very gradually. But with their flexibility they did not recover action, so that they were really dead. Till this time I had imagined that it might be possible to prolong life to any period by freezing a person in the frigid zone, as I thought all action and waste would cease until the body was thawed. I thought that, if a man would give up the last ten years of his life to this kind of alternate oblivion and action, it might be prolonged to a thousand years; and, by getting himself thawed every hundred years, he might learn what had happened during his frozen condition. Like other schemers, I thought I should make my fortune by it; but this experiment undeceived me." (P. 284.)

CHARITY HOSPITAL, MEDICAL LIBRARY.

The present chapter abounds in the detail of numerous experiments, upon both man and animals, made with a view to determine the vital capability of developing heat. Those which relate to this faculty in the hybernating species possess much interest, as also those which have reference to the power of resistance to the influence of an external temperature, above the natural standard of the body.

The chapter on animal heat forms the last of that part of the work which is strictly *physiological*, and the six ensuing comprise its *pathological* division. Mr. Hunter commences by remarking that, to understand the imperfections or diseases of the body, "it is necessary, in the first place, to describe its perfect or healthy state;" thus recognizing the principle that physiology is the only sure foundation of the whole science of medicine; and towards the adoption of which principle by all enlightened cultivators of our art, his own labours so eminently contributed. Its elucidation forms the material of all his pathological remarks, which, it were almost unnecessary to say, have their principal application to surgery. Any attempt, however, at analysis, within moderate limits, would be very imperfect; and hence we shall select only particular portions which appear to exemplify the state of pathology at this era, and the mode also in which the subject is discussed in these lectures.

The chapter upon "Sympathy" contains some excellent illustrations of its phenomena. Mr. Hunter, having been engaged in the discussion of what he had regarded as "primary susceptibility of disease," by which was understood the disposition, in any structure, to assume a morbid action, in consequence of the direct application of some exciting cause, proceeds to speak of what he designates a "secondary susceptibility." This, he observes, arises from sympathy, by which "an action arises without any immediate impression in a secondary way, either acting in conjunction with the part immediately impressed, or taking the whole action on itself." Various modes of sympathy are ranked under distinct heads: thus, he speaks, in the first place, of the sympathy of "a local with a local disease," and of an "universal with a local," holding that all sympathies must arise from a local cause. Another classification of sympathies, which he makes, possesses somewhat more importance; it is into the natural and the diseased: and here follow some remarks which we shall insert at length, on account of their own excellence, as well as of their characteristic style.

"Sympathy may be divided into two kinds, the natural and the diseased. The diseased is when sound parts sympathise with the diseased, and probably the diseased with the diseased, which is what I mean to explain. The sympathy of one diseased part with the diseases of another part will include the idea of revulsion, as revulsion consists in the production of a disease in one part to cure a disease in another part; which shows that this one part, while under disease, can be affected by a diseased action being produced in another part, or the cessation of one action in consequence of another having taken place in another part. Natural sympathy takes place more readily, and its actions are more strongly marked, in proportion as the powers of the machine are capable of repairing an injury received. On the other hand, it takes place more slowly, and is less evident, as the powers of life are more languid. In many diseased states, the condition of the whole body is often such that it more readily falls into sympathy at one time than at others. Thus, we find people at particular periods much affected by slight causes, while at other times considerable mischief received will hardly affect them. Some people are naturally more readily affected than others, as will be evident in disease. Sympathy sometimes proves fatal, as in children from teething. But this depends, in great measure, on the parts sympathizing, or the number of parts that sympathize. Sympathies are often not reciprocal: the liver never sympathizes with the shoulder, nor the urethra with the testis; nor, when the glans penis is affected, does any irritation pass to the bladder; but often they are, as, for example, between the head and the stomach. Sympathies are generally simple; we hardly ever find two parts sympathizing with the same cause: however, the spasmodic convulsion of both hands, or hands and feet, &c., as sometimes takes place, may be called a double sympathy. Sympathy is common and uncommon. The first is where it takes place more readily between some parts than it does between others, as between the stomach and head, the stomach and the skin, the testes and urethra. Sympathy may be called uncommon when parts sympathize with diseased parts that were never known to sympathize in health. A gentleman had a sore on the inside of his thigh, which itched so intolerably that he could not avoid scratching it, and, when he did, it always produced tightness in his chest and shortness of breathing, which he never had but at these times. Lord Cavendish's father always felt pain in the left arm from a stone in the bladder: this pain was the only indication of a want to make water." (P. 320.)

In the next chapter we have some very interesting observations on the general nature of diseases, which are divided into constitutional, local, and mixed. The doctrines which are here advanced are such as, with some slight exceptions, will be assented to in the present day; and we think that Hunter, in the present chapter, shows himself decidedly in advance of his own times, in the pathological remarks which he adduces, and more especially in the views which he offers upon the reciprocal influence existing, in many cases, between local and constitutional maladies; wherein he maintains and illustrates the doctrine of the operation of general disease in the development of local affections, and of the modifications which wounds, or other local derangements, undergo, in consequence of constitutional peculiarities with which they may happen to be associated. Generally speaking, however, there is nothing in these observations which would command any very special regard in the present age, the whole question having, of late years, been so maturely considered, particularly since the appearance of Mr. Abernethy's work "On the Constitutional Origin and Treatment of Local Diseases."

Inflammation and its consequences are treated of in three chapters, and receive, as might be expected, very full discussion; but any sketch of the doctrines propounded, or any detail of the experiments by which they are supported, will fall more properly under notice when the separate treatise on this topic comes under review; at present, we can only state that Hunter enters freely into the matter in the present volume, adducing, however, in illustration of his views, comparatively few of the facts or experiments on which they rest.

The last nine chapters of the volume before us are given to the practical illustration of the principles previously enounced; and, in these, we have some of the principal diseases, falling under the management of the surgeon, explained, illustrated, and their treatment described. Our limits will permit us only to make one or two references; and, for this purpose, we shall select the sections embracing "Injuries of the Head" and "Aneurism." The former topic does not occupy that amount of space, in the text, which its high importance would seem to demand; but the reader is less disposed to be dissatisfied on this account, as the Editor's marginal notes are exceedingly copious, and exhibit the condition of this branch of surgery in accordance with the principles and practice of the most experienced surgeons of the present day.

Mr. Hunter considers the effects of injuries of the head, mainly, under the popular divisions of concussion and compression. The detail which he affords of the symptoms of these cerebral conditions is, in general, precise and accurate, and such as corresponds with daily observation. When, however, he comes to speak of the treatment of these affections, we are strongly reminded of the great improvements which have been wrought in modern surgery, and most especially as regards the employment of the trephine. Hunter justifies the employment of the trephine, in cases of mere fissure, as the operation *can do no harm;* in cases of ascertained depression of the outer table only, since there can be no certainty regarding the state of the inner table, and neglect to operate might produce mischief hereafter; in young children, in whom depression exists without symptoms of compression, with the same fear of future consequences; and, lastly, in all cases of violence attended with compression, even in the absence of fracture, wherein he declares the operation "somewhere or other" to be absolutely necessary. It is almost superfluous to remark upon the immense advance which has, of late years, been made in the management of such cases; in some of which, the operation would be deemed altogether unjustifiable, and in none would it be deemed applicable without some grave and important reasons, and after the failure of other measures.

The improvement, wrought by Hunter, in the surgical treatment of Aneurism, is one of those facts with which every one in the profession must be familiar. The practice of surgery, in this branch, was, previously to his day, in a very imperfect condition. This cannot be better shewn than by inserting the following passage upon the treatment of aneurism, in which he combats the particular views of Bromfield and Pott; and, as it also offers an excellent illustration of the style and manner adopted in the lectures which treat on practical points, we give it at full length:

"Mr. Bromfield objects to every operation, either amputation, or for the aneurism; this would be just if what he asserts was true, viz. that the whole of the arterial system is in general diseased, which however is certainly not the case. He says, too, 'that the injecting of parts in dead bodies having shown that in particular subjects the branches sent off have now and then formed anastomoses with other branches given off lower down, has led to very extravagant notions of the smaller branches being always able to carry on the circulation; and an extravagant proposition has been suggested by some people to tie up the principal trunk of an artery in the extremities. I once saw an attempt of this kind in a true aneurism of the ham, in which I shall only remark that the patient died; and I do believe that the embarrassments which occurred, as well as the event of the operation, will deter the gentleman' (meaning me) 'who performed it from making a second attempt in a similar case.' Now unfortunately either for Mr. Bromfield or myself, this is the very case from which I have formed favorable ideas of the success of future operations of a similar nature. Mr. Pott, after describing the disease in its last and most violent stage, just preceding dissolution, and when it has done all the mischief it can do without destroying the life of the patient, says, "If a man was to be asked how the disease was to be treated, he would answer, from theory, that the artery should be tied above and below the tumor, and the coagulated blood be evacuated; but that the artery is generally diseased some way above the dilatation, especially the popliteal.' He also observes, 'that the want of collateral branches of sufficient size to carry on the circulation is another powerful impediment to the operation.'

"When the aneurism has arrived at the stage which Mr. Pott describes, perhaps the only thing is to amputate above the dilated part of the artery; but Mr. Pott should have considered, that before these threatening symptoms there is a stage when all the surrounding parts are sound. If this be true, would any man allow a disease in a part to go on till the surrounding parts are diseased and past cure?

"The events of all diseases are of two kinds: first, where the termination is certain; 2d, where it is dubious. The aneurism is of the first kind; its event is certain. Now I do aver, then, that there is a stage of the disease in which the operation is safe. I do not, certainly, know how to judge of this state from the external appearance, but from what I have seen of aneurisms, I believe it will allow of considerable latitude. My opinion is, that the operation should be performed, 1st, when the disease has done no mischief to the surrounding parts; 2d, where it is distinct and circumscribed, not connected with parts which may not be curable when exposed, as bones; 3dly, when there is a distinct pulsation. How early the operation may be performed I do not certainly know, but my opinion is that it may be done as soon as the aneurism is known to exist. By some it has been recommended to permit the disease to exist some time first, because, say they, as the circulation becomes obstructed, a freer communication will take place between the branches above and below; but this would not be until the obstruction had subsisted some time, and I would not wait for this, for fear of the consequences described by Mr. Pott.

"When the disease is in an advanced stage, I agree with Mr. Pott in thinking amputation necessary and preferable, but not under the circumstances above mentioned. The earlier, therefore, the operation for the aneurism is performed the better, not waiting with the expectation that an increased size of the aneurism will produce an increased size of the collateral branches. That the popliteal artery, according to Mr. Pott, is oftener diseased above the aneurism than other arteries, I cannot well determine, but can see no reason why it should be so. If the artery, however, cannot be tied above the aneurism in the operation, where can it be tied if the limb be amputated? Why not tie it up higher in the sound parts, where it is tied in amputation, and preserve the limb? The circumstances to be regarded chiefly turn upon the collateral branches being sufficient to carry on the circulation. The only branches which admit of this question are the popliteal, femoral, and brachial; the other arteries either having a very free anastomosis, or being out of the way of any operation.

"In this account it may be supposed that I carry my notions too far; but it is to be understood that I only give my own feelings upon this subject, and I go no further in theory than I would perform in practice, if patients, being acquainted with the consequences of the disease, would submit to, or rather desire, the operation; nor do I go further than I now think I would have performed on myself were I in the same situation. Not that I would have it supposed that I would recommend this at large: I would have no one perform an operation that he is not clear about the propriety of himself, especially when it requires more anatomical skill than falls to the share of most practitioners." (P. 647.)

In giving directions for the operation, Mr. Hunter does not, in the present volume, describe the one which usually goes by his name, and the introduction of which into practice will ever constitute one of his strongest claims to the gratitude of mankind, that of tying the artery at a distance from the tumour, and between it and the heart,—but he directs that which was most commonly in use previously. The reason of this is explained by the Editor, in a marginal note, from which it appears, by a comparison of dates, that this lecture was only delivered about a year after the first performance of this latter operation; and it seems that, with his wonted philosophical caution, he did not yet think himself justified in pronouncing definitely upon its superiority.

We cannot bring our notice of the present volume to a close without offering our testimony to the admirable way in which the editor and annotator has fulfilled his part of the undertaking. The advancement and improvements that have been effected, up to our own day, not only in practical surgery, but in all the collateral departments, are constantly brought before the reader's attention in clear and concise terms. The errors of expression also, into which Hunter was apt to fall, are judiciously pointed out, and rectified where, from want of precision, the student might be led into error. Indeed, taking these lectures in conjunction with the notes, we hardly know any work that will be read with greater advantage. The student may obtain from it an excellent initiation in the principles of his future profession; the surgeon will receive many excellent hints which he may readily discover opportunities of applying in practice; and the physician may gather from its contents just and philosophical notions of the true theory of the whole art of healing, which cannot fail to heighten his devotion to its pursuit. We think, moreover, that no one will finish the perusal of this publication, without some feeling of honorable anxiety to imitate the virtues and labours of John Hunter, who not only now, but probably in all future time, must be regarded as the pride and boast of British surgery.

